# Lysophosphatidylcholine acyltransferase 1 controls mitochondrial reactive oxygen species generation and survival of retinal photoreceptor cells

**DOI:** 10.1016/j.jbc.2022.101958

**Published:** 2022-04-20

**Authors:** Katsuyuki Nagata, Daisuke Hishikawa, Hiroshi Sagara, Masamichi Saito, Sumiko Watanabe, Takao Shimizu, Hideo Shindou

**Affiliations:** 1 Department of Lipid Signaling, National Center for Global Health and Medicine, Shinjuku-ku, Tokyo, Japan; 2 Medical Proteomics Laboratory, Institute of Medical Science, The University of Tokyo, Minato-ku, Tokyo, Japan; 3 Department of Biochemistry and Molecular Biology, Faculty of Medicine, The University of Tokyo, Bunkyo-ku, Tokyo, Japan; 4 Division of Molecular and Developmental Biology, Institute of Medical Science, The University of Tokyo, Minato-ku, Tokyo, Japan; 5 Department of Lipid Science, Graduate School of Medicine, The University of Tokyo, Bunkyo-ku, Tokyo, Japan

**Keywords:** photoreceptor cells, retinal degeneration, membrane phospholipids, saturated fatty acid, apoptosis, ANOVA, analysis of variance, Crb1, Crumbs homolog 1, DEGs, differentially expressed genes, DHA, docosahexaenoic acid, DPPC, dipalmitoyl PC, ER, endoplasmic reticulum, FA, fatty acid, GCL, ganglion cell layer, GO, gene ontology, HZ, heterozygous, IEGs, immediate early genes, INL, inner nuclear layer, KO, knockout, LPC, lysophosphatidylcholine, LPCAT1, lysophosphatidylcholine acyltransferase 1, LPLATs, lysophospholipid acyltransferases, MRM, multiple reaction monitoring, ONL, outer nuclear layer, OS, outer segment, P8, postnatal day 8, PC, phosphatidylcholine, PDE6, phosphodiesterase 6, PUFA, polyunsaturated FA, rd, retinal degeneration, ROS, reactive oxygen species, RPE, retinal pigment epithelial, SCD, stearoyl-CoA desaturase, SFA, saturated FA, SM, sphingomyelin, TEM, transmitted electron microscopy, TUNEL, terminal deoxynucleotidyl transferase dUTP nick-end labeling

## Abstract

Due to their high energy demands and characteristic morphology, retinal photoreceptor cells require a specialized lipid metabolism for survival and function. Accordingly, dysregulation of lipid metabolism leads to the photoreceptor cell death and retinal degeneration. Mice bearing a frameshift mutation in the gene encoding lysophosphatidylcholine acyltransferase 1 (*Lpcat1*), which produces saturated phosphatidylcholine (PC) composed of two saturated fatty acids, has been reported to cause spontaneous retinal degeneration in mice; however, the mechanism by which this mutation affects degeneration is unclear. In this study, we performed a detailed characterization of LPCAT1 in the retina and found that genetic deletion of *Lpcat1* induces light-independent and photoreceptor-specific apoptosis in mice. Lipidomic analyses of the retina and isolated photoreceptor outer segment (OS) suggested that loss of *Lpcat1* not only decreased saturated PC production but also affected membrane lipid composition, presumably by altering saturated fatty acyl-CoA availability. Furthermore, we demonstrated that *Lpcat1* deletion led to increased mitochondrial reactive oxygen species levels in photoreceptor cells, but not in other retinal cells, and did not affect the OS structure or trafficking of OS-localized proteins. These results suggest that the LPCAT1-dependent production of saturated PC plays critical roles in photoreceptor maturation. Our findings highlight the therapeutic potential of saturated fatty acid metabolism in photoreceptor cell degeneration–related retinal diseases.

In addition to the *de novo* pathway (Kennedy pathway) of phospholipid biosynthesis (1), fatty acyl moieties of membrane phospholipids are turned over dynamically by deacylation and reacylation cycles, which is called the Lands’ cycle (2). Appropriate regulation of phospholipid composition by the Lands cycle is required for various cellular functions, including lipoprotein production and lipid mediator production (3). In the Lands cycle, lysophospholipid acyltransferases (LPLATs) involve fatty acid (FA) reacylation to generate membrane phospholipid diversity. Depending on their substrate (lysophospholipids and acyl-CoAs) selectivity, LPLATs produce specific types of membrane phospholipids, such as polyunsaturated FA (PUFA)–containing phospholipids and saturated FA (SFA)–containing phospholipids.

Among LPLATs, lysophosphatidylcholine acyltransferase 1 (LPCAT1, also known as LPLAT8) produces saturated phosphatidylcholine (PC) using lysophosphatidylcholine (LPC) and saturated fatty acyl-CoA, such as palmitoyl-CoA (4, 5, 6, 7). Previous studies have shown that the LPCAT1-mediated production of saturated PC is required for the proper functioning of pulmonary surfactant in the lung (7, 8) and trafficking of growth factor receptors in cancer cells (9). In addition, *Lpcat1* is essential for the survival of retinal photoreceptor cells (10, 11). The critical role of *Lpcat1* in photoreceptor cells was originally uncovered by the analysis of retinal degeneration 11 (*rd*11) mice, possessing a frameshift mutation in the *Lpcat1* gene. In *rd*11 mice, the retinal outer nuclear layer (ONL) composed of photoreceptor cells is rapidly diminished, causing vision loss until 1 month of age.

Photoreceptor cells possess a unique membrane phospholipid composition (12). In the membrane of photoreceptor cells, especially in the outer segment (OS) discs, docosahexaenoic acid (DHA)–containing phospholipids are extremely enriched. Our recent study revealed that the loss of DHA-containing phospholipids leads to the collapse of the OS disc structures and retinal degeneration (13). In parallel with this, significant levels of SFA-containing phospholipids were also present in photoreceptor cells. Therefore, the photoreceptor degeneration in *rd*11 suggests that the LPCAT1-mediated production of SFA-containing phospholipids contributes to photoreceptor survival. However, the molecular basis underlying retinal degeneration in *rd*11 mice remains unclear.

In this study, we investigated the role of LPCAT1 in the retina using the *Lpcat1* knockout (KO) mice. Consistent with *rd*11 mice, the retina of *Lpcat1* KO mice showed a rapid loss of ONL with decreased levels of dipalmitoyl PC (DPPC), the major saturated PC species in the retina. We demonstrated that retinal degeneration in *Lpcat1* KO mice was caused by apoptosis of photoreceptor cells, which occurs in a light-independent manner. Furthermore, *Lpcat1* KO photoreceptor cells accumulated mitochondrial reactive oxygen species (ROS). Our study demonstrated that the roles of LPCAT1 are not only in DPPC production for maintenance of membrane integrity but also in the regulation of normal mitochondrial functions.

## Results

### Photoreceptor cell apoptosis in *Lpcat1* KO mice

**Figure 1:**
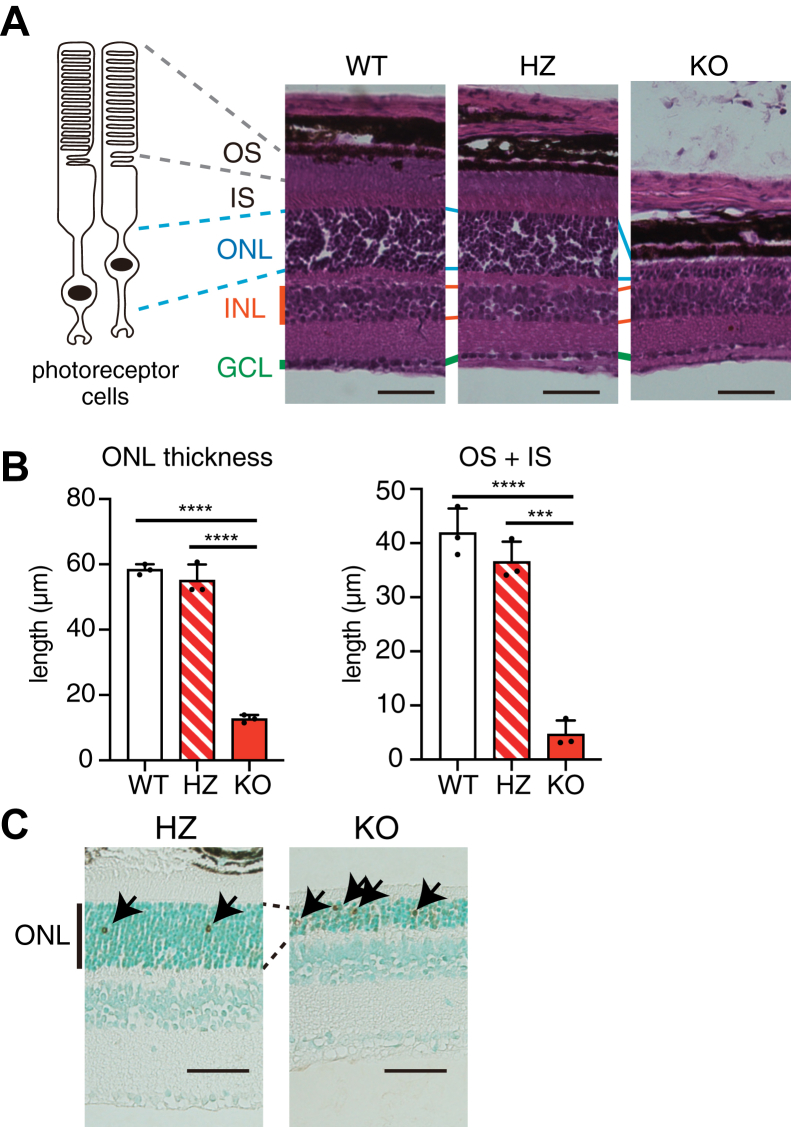
**Severe retinal degeneration in *Lpcat1* knockout (KO) mice.***A*, histology of *Lpcat1* wildtype (WT), heterozygous (HZ), and KO littermate mice retina at 6-week-old (n = 3, for each group). Retinal tissue sections were stained with hematoxylin and eosin. Representative images from three independent experiments were shown. The *left images* illustrate the structure of photoreceptor cells. Scale bars are 50 μm. *B*, the bar graphs showed the outer nuclear layer (ONL) thickness (*left panel*; n = 3, for each group) and the sum of the length of outer segment (OS) and inner segment (IS) (*right panel*; n = 3, for each group). Significance is based on one-way ANOVA followed by Bonferroni’s multiple comparisons test (∗∗∗*p* < 0.001, ∗∗∗∗*p* < 0.0001). Data are shown as mean + SD of independent experiments. *C*, representative images of terminal deoxynucleotidyl transferase dUTP nick-end labeling (TUNEL) staining of 4-week-old *Lpcat1* HZ and littermate KO retina (n = 3, for each group). Nuclei were stained with methyl green. *Arrows* indicate TUNEL-positive nuclei detected by 3,3′-diaminobenzidine (DAB, shown in *brown*). Representative images from three independent experiments are shown. Scale bars are 50 μm. GCL, ganglion cell layer; INL, inner nuclear layer.

A previous study has shown that a frameshift mutation in the *Lpcat1* gene in *rd*11 and B6-JR2845 mice leads to the spontaneous development of severe retinal degeneration (10). Therefore, to investigate whether the loss of function of LPCAT1 causes retinal degeneration, we used mice genetically deleted for *the Lpcat1* gene. Consistent with *rd*11 and B6-JR2845 mice, a dramatic reduction in ONL and OS + inner segment thickness was observed in 6-week-old *Lpcat1* KO mice compared to that in *Lpcat1* wildtype (WT) and heterozygous (HZ) mice (Fig. 1, *A* and *B*). Meanwhile, the thickness of the inner nuclear layer and ganglion cell layer were similar among the three groups (Fig. 1, *A* and *B*). The histological differences between *Lpcat1* WT and HZ mouse retinas were not observed.

Apoptosis of photoreceptor cells is a major cause of retinal degeneration (14). To assess the cause of retinal degeneration in *Lpcat1* KO mice, we visualized the apoptotic cells in the retina using terminal deoxynucleotidyl transferase dUTP nick-end labeling (TUNEL). TUNEL analyses indicated that the number of apoptotic photoreceptor cells was higher in 4-week-old *Lpcat1* KO mice than in HZ mice (Fig. 1*C*). Notably, the increase in apoptotic cells in global *Lpcat1* KO mice was not observed in the lungs, liver, or other retinal cell layers (Figs. 1*C* and S1, *A* and *B*). These results indicate that loss of *Lpcat1* leads to photoreceptor cell apoptosis in a specific manner.

### Onset of the photoreceptor apoptosis in *Lpcat1* KO mice

**Figure 2:**
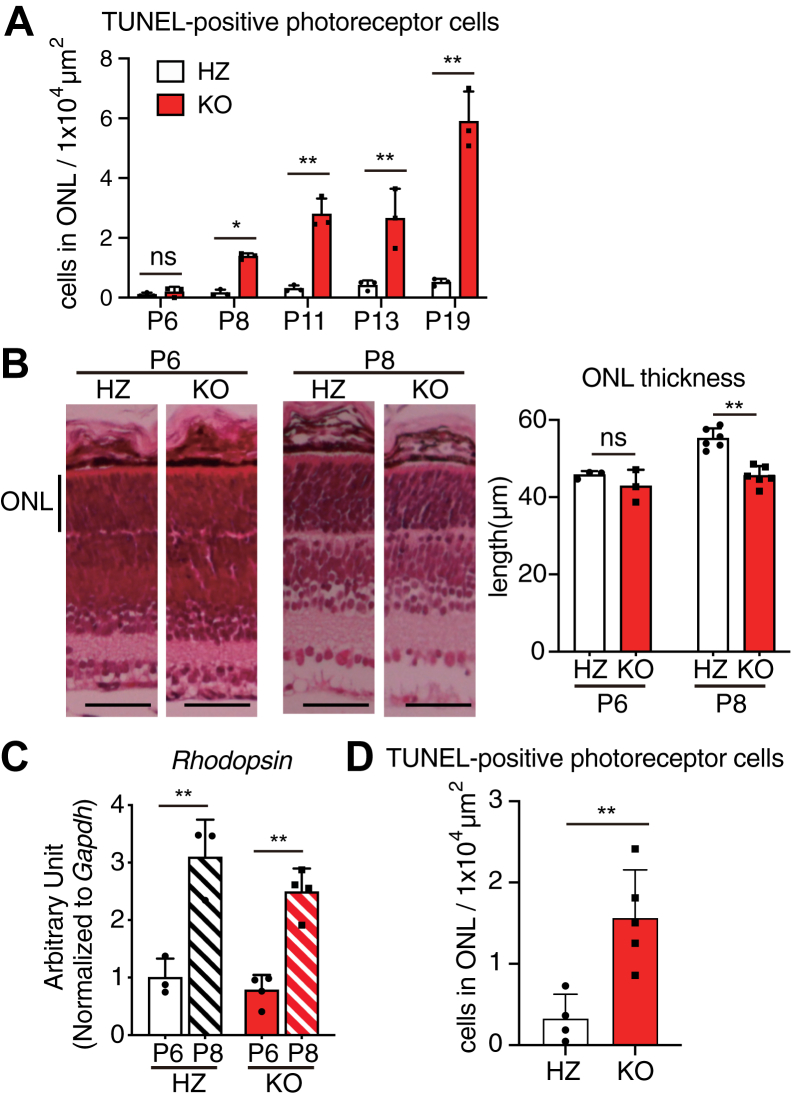
**Onset of photoreceptor apoptosis in *Lpcat1* knockout (KO) mice.***A*, the average value of TUNEL-positive cells per outer nuclear layer (ONL) areas in the retinal section of postnatal day 6 (P6), P8, P11, P13, and P19 *Lpcat1* heterozygous (HZ) and KO (n = 3 for each group). *B*, the ONL thickness in P6 and P8 *Lpcat1* HZ and littermate KO mice. The *left images* showed the retinal structure stained with hematoxylin and eosin. Representative images from over three independent experiments were shown. The *right bar graph* showed the ONL thickness (P6, n = 3 for each group; P8, n = 6 for each group). Scale bars are 50 μm. *C*, the levels of rhodopsin mRNA in P6 and P8 of *Lpcat1* HZ and littermate KO retina. The expression level was normalized by glyceraldehyde 3-phosphate dehydrogenase (*Gapdh*) (n = 3 for HZ, n = 4 for KO). *D*, the average value of TUNEL-positive cells per outer nuclear layer (ONL) areas in P9 dark-reared *Lpcat1* HZ and littermate KO retina (n = 4 for HZ, n = 5 for KO). *A*–*C*, significance is based on two-way ANOVA followed by Bonferroni’s multiple comparisons test (∗*p* < 0.05, ∗∗*p* < 0.01, ns; no significance). Data are shown as mean + SD of independent experiments. *D*, significance is based on the unpaired *t* test (∗∗*p* < 0.01). Data are shown as mean + SD of independent experiments. TUNEL, terminal deoxynucleotidyl transferase dUTP nick-end labeling.

To assess the onset of photoreceptor cell apoptosis in *Lpcat1* KO mice, we performed TUNEL staining of the retina at different time points. Apoptotic cells in the retina of *Lpcat1* KO mice increased greatly from postnatal day 8 (P8) (Fig. 2*A*). Consistent with this, the thickness of the ONL was thinner in *Lpcat1* KO mice at P8, while no difference in the apoptotic cell number and ONL thickness was seen in the P6 retina (Fig. 2, *A* and *B*). Dramatic increases in the expression of *rhodopsin*, critical for phototransduction and OS formation (15), at this time point (Fig. 2*C*), suggests that photoreceptor apoptosis in *Lpcat1* KO retinas occurred concomitantly with photoreceptor OS formation.

As OS is required for phototransduction, we hypothesized that photoreceptor cell death in *Lpcat1* KO mice is related to light exposure. Thus, we investigated the apoptosis of photoreceptor cells in P9 mice, which were raised in complete darkness from P2 to P9. However, photoreceptor cell apoptosis was observed even in dark-reared *Lpcat1* KO mice (Fig. 2*D*), suggesting that retinal degeneration in *Lpcat1* KO mice was independent of light stimulation.

### Altered phospholipid composition in *Lpcat1* KO mouse retina

**Figure 3:**
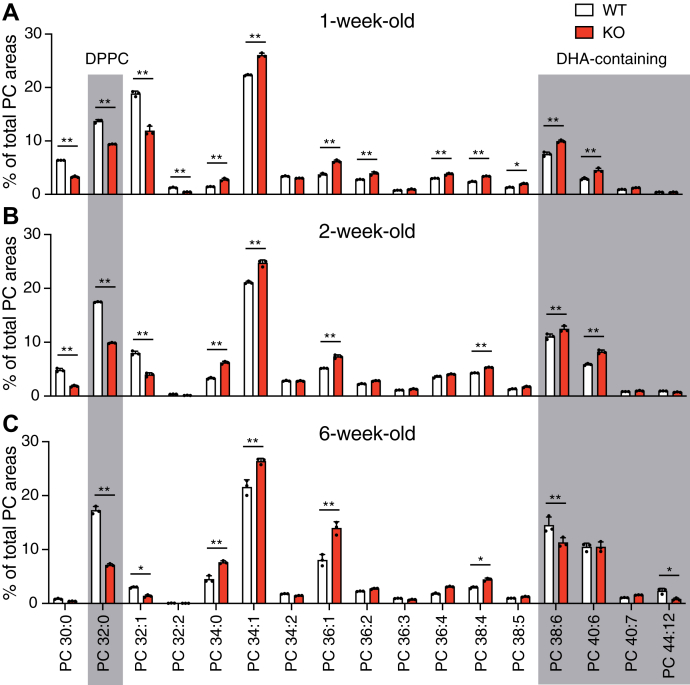
**Loss of LPCAT1 leads to the alteration of the retinal phosphatidylcholine (PC) composition.** PC composition of the retina. *A*–*C*, retinas were prepared from 1- (*A*), 2- (*B*), and 6-week-old (*C*) *Lpcat1* wildtype (WT) and knockout (KO) mice (n = 3 for each group). Significance is based on two-way ANOVA followed by Bonferroni’s multiple comparisons test (∗*p* < 0.05, ∗∗*p* < 0.01). Data are shown as mean + SD of independent experiments.

Photoreceptor cells undergo dynamic morphological and metabolic alterations during retinal maturation (16). Since LPCAT1 produces SFA-containing PC species, we next analyzed PC composition and LPCAT1 expression during retinal maturation. As shown in Figure 3, liquid chromatography-tandem mass spectrometry (LC-MS/MS) analysis showed a gradual alteration in retinal PC composition during maturation. Consistent with previous studies (12, 13), the proportion of DHA-containing PC species, including PC 38:6, PC 40:6, and PC 44:12, increased along with retinal maturation (Fig. 3). Coinciding with the onset of photoreceptor cell apoptosis in *Lpcat1* KO mice, DPPC, a major product of LPCAT1, was increased in the retina of WT mice between 1 and 2 weeks of age (Fig. 3, *A* and *B*). Consistent with the substrate selectivity of LPCAT1 *in vitro* (5, 7), DPPC levels were decreased by half in *Lpcat1* KO retinas compared to WT retinas. Thus, DPPC production in the retina largely depends on LPCAT1 expression. However, because the induction of *Lpcat1* mRNA and protein was not observed during retinal maturation (Fig. S2, *A* and *B*), post-translational modifications or the increased substrate supply for LPCAT1, such as LPC and palmitoyl-CoA, may affect the age-dependent elevation of DPPC.

### Transcriptomic analysis of *Lpcat1* KO retina and isolated photoreceptor cells

**Figure 4:**
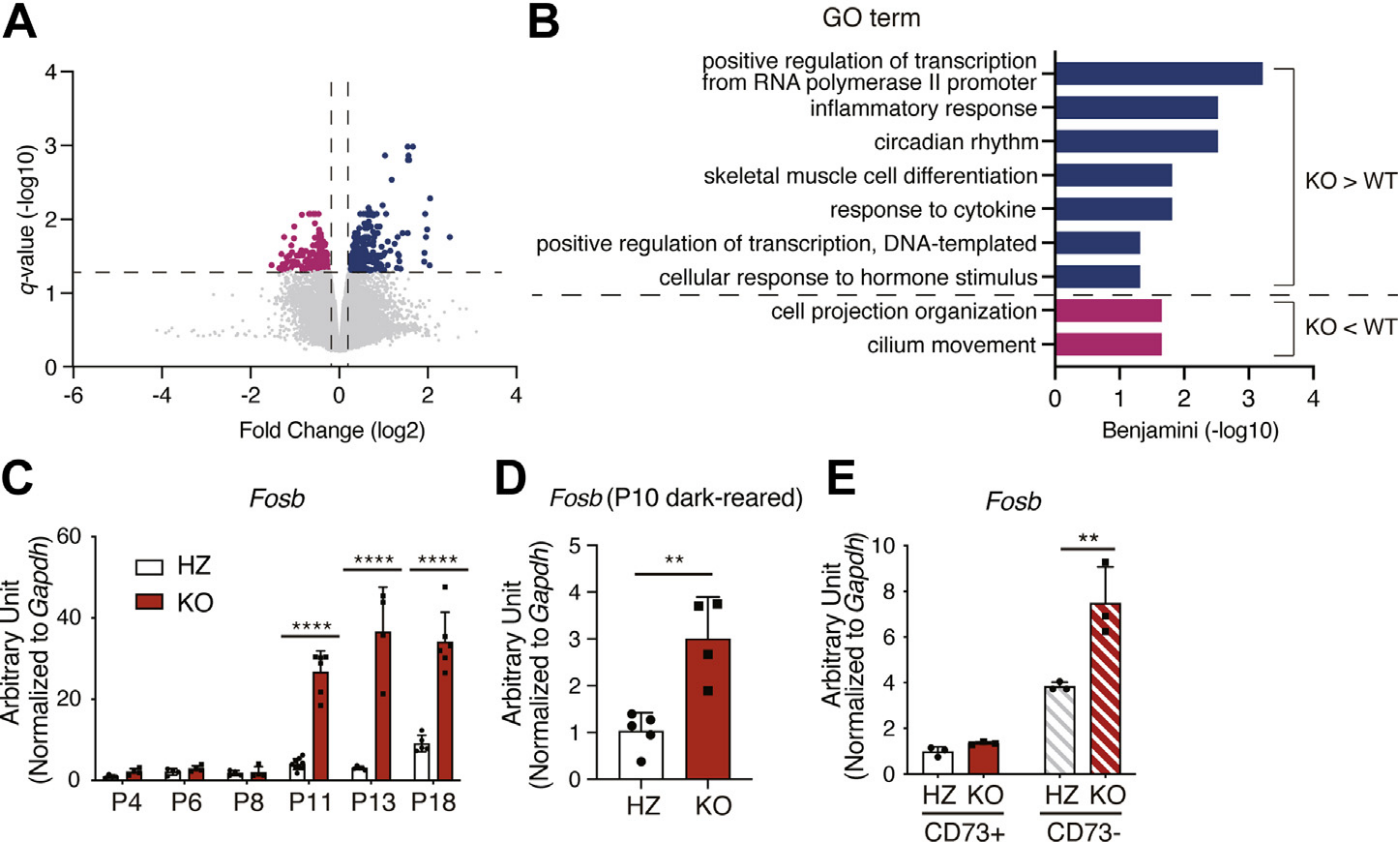
**Transcriptomic analysis of *Lpcat1* knockout (KO) retina.***A*, volcano plot of differentially expressed genes (DEGs) between postnatal day 14 (P14) *Lpcat1* wildtype (WT) and KO retina. DEGs (fold change >1.2 or <−1.2, q-value < 0.05) increased in *Lpcat1* KO are highlighted in *blue*, and DEGs decreased in *Lpcat1* KO are highlighted in *magenta* (n = 4 for each group). *B*, functional annotation of upregulated or downregulated genes (shown in *blue* or *magenta* in *A*) in *Lpcat1* KO retina. *C*–*E*, the mRNA expression of *Fosb* during neonatal development (*C*, n = 4 for HZ and n = 9 for KO), in P10 dark-reared littermates (*D*, n = 4 for HZ and n = 5 for KO), and in CD73-positive (photoreceptor cells) or CD73-negative cells (*E*, n = 3 for each group) of *Lpcat1* heterozygous (HZ) and KO retina. The expression level was normalized by glyceraldehyde 3-phosphate dehydrogenase (*Gapdh*). Data are shown as mean + SD of independent experiments. *C* and *E*, significance is based on two-way ANOVA followed by Bonferroni’s multiple comparisons test (∗∗*p* < 0.01, ∗∗∗∗*p* < 0.0001). *D*, significance is based on unpaired *t* test (∗∗*p* < 0.01).

Next, we aimed to clarify the cause of photoreceptor cell death in *Lpcat1* KO mice by comparing the mRNA expression profiles with the control mice. First, we analyzed the transcriptomic differences in the 2-week-old retinas of *Lpcat1* WT and KO mice (Fig. 4, *A* and *B*). Gene groups that increased significantly in *Lpcat1* KO retinas were analyzed by functional classification using “the Database for Annotation, Visualization, and Integrated Discovery.” These results suggest that many of the genes upregulated in *Lpcat1* KO retinas were related to immune and inflammatory responses and genes downregulated in *Lpcat1* KO retinas were involved in the cell projection organization and cilium movement (Fig. 4*B* and Table S1). In addition, we found that the expression of genes termed immediate early genes (IEGs), such as *Fosb*, *Fos*, *Egr1*, and *Egr2*, was higher in *Lpcat1* KO than in the control retina (Table S1). Among them, we found that *Fosb* expression in *Lpcat1* KO was significantly higher than in the control retinas, even in dark-reared mice (Fig. 4, *C* and *D*). As IEGs are rapidly upregulated by various cellular stimuli, particularly in neurons (17), we performed qPCR analyses using isolated a photoreceptor cell marker CD73-positive and CD73-negative cells (18) from the retina to identify the cellular sources of their upregulation (Fig. S3*A*). The qPCR results showed increased expression of IEGs in CD73-negative cells (Figs. 4*E* and S3*B*). The induction of *Fosb* in *Lpcat1* KO retinas was slightly delayed from the onset of retinal degeneration (Fig. 4*C*), suggesting that it might be triggered by apoptosis of photoreceptor cells.

To clarify the primary cause of photoreceptor cell apoptosis in *Lpcat1* KO mice, we performed microarray analysis using isolated photoreceptor cells at P8 when apoptosis of photoreceptor cells in *Lpcat1* KO mice begins. As a result of functional classification analysis, no significant enrichment of the biological process altered in *Lpcat1* KO photoreceptor cells was found in the gene list, except for the categories of cell adhesion (Fig. S3, *C* and *D*). Since most of the downregulated genes listed in these categories in *Lpcat1* KO photoreceptor cells were reported to be highly expressed in retinal pigment epithelial (RPE) cells, it may reflect the reduced contamination of RPE cell fragments in isolated photoreceptor cells in *Lpcat1* KO mice. Consistently, qPCR analysis showed that mRNA expression of RPE65, an RPE-specific gene, was lower in isolated photoreceptor cells of *Lpcat1* KO mice than in control mice (Fig. S3*E*). Although the cause of this observation is currently unknown, transcriptomic analysis of isolated photoreceptor cells suggested that loss of *Lpcat1* did not induce the obvious changes in the gene expression profiles in photoreceptor cells.

A previous study reported that the failure of SFA-containing PC production due to loss of LPCAT1 leads to excessive PUFA accumulation and endoplasmic reticulum (ER) stress response in the retina (19). However, under the present assay conditions, no induction of CCAAT-enhancer-binding protein homologous protein, an ER stress marker, was observed both in *Lpcat1* KO retina and isolated photoreceptor cells (Fig. S3*F*).

### Trafficking and functions of OS-localized proteins in *Lpcat1* KO mice

**Figure 5:**
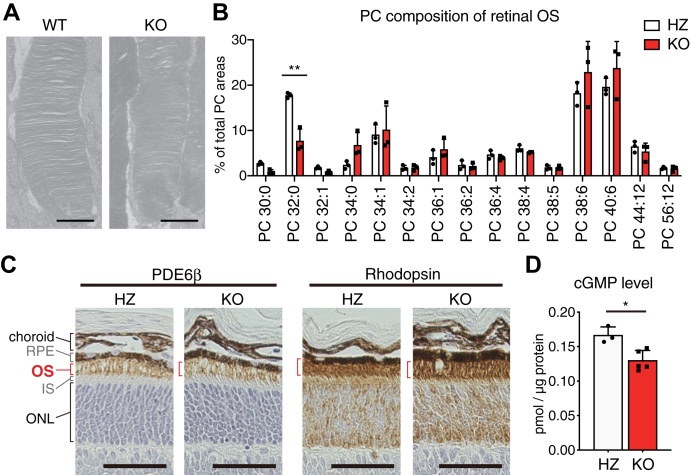
**Effect of LPCAT1 deficiency on the structure and function of photoreceptor cells.***A*, representative transmission electron microscopy (TEM) images of photoreceptor outer segment (OS) in 4-week-old *Lpcat1* knockout (KO) and littermate wildtype (WT) mice (n = 3, for each group). Representative images from three independent experiments were shown. Scale bars are 1 μm. *B*, fatty acid composition of phosphatidylcholine (PC) in the isolated photoreceptor OS. OS samples were prepared from postnatal day 19 (P19) *Lpcat1* heterozygous (HZ) and KO mice (n = 3 for each group). Significance is based on two-way ANOVA followed by Bonferroni’s multiple comparisons test (∗∗*p* < 0.01). Data are shown as mean + SD of independent experiments. *C*, representative images of PDE6β and rhodopsin in P13 *Lpcat1* HZ and littermate KO mice by immunohistochemistry analysis. Signals were detected by 3,3′-diaminobenzidine (DAB, shown in *brown*), observed in the OS region (shown in *red brackets*). The *dark brown colors* in RPE and choroid were DAB-independent (n = 3 for each group). Representative images from three independent experiments were shown. Scale bars are 50 μm. *D*, cGMP level in P11 *Lpcat1* HZ and littermate KO retina (n = 3 for HZ, n = 5 for KO). Data are shown as mean + SD of independent experiments. Significance is based on unpaired *t* test (∗*p* < 0.05). IS, inner segment; ONL, outer nuclear layer; OS, outer segment; RPE, retinal pigment epithelium.

Based on the microarray analyses showing no obvious causative changes in photoreceptor cell death in *Lpcat1* KO mice, we investigated whether the altered membrane phospholipid composition in *Lpcat1* KO photoreceptor cells influences the localization and functions of proteins essential for photoreceptor survival. Consistent with the previous observation of *Lpcat1* mutant mice (10), the structure of *Lpcat1* KO OS discs photoreceptor cells appeared normal (Fig. 5*A*). We then explored the possibility that an altered PC composition of *Lpcat1* KO photoreceptor OS membrane affected the localization and function of phototransduction-related proteins since the mislocalization of photoreceptor OS-localized proteins, such as rhodopsin and phosphodiesterase 6β (PDE6β), is observed in various types of retinal degeneration (20, 21). In parallel with the DHA-containing PCs enriched in the OS discs (13), we found that DPPC was also abundant in the OS and decreased by half in *Lpcat1* KO (Fig. 5*B*). This result suggested that the LPCAT1-mediated production of DPPC contributes to maintaining proper PC composition in the photoreceptor OS membrane.

As we found that *Lpcat1* deletion leads to altered OS membrane PC composition, we next investigated the localization of rhodopsin and PDE6β in the retina. Unexpectedly, both rhodopsin and PDE6β were normally localized in the photoreceptor OS in *Lpcat1* KO mice, despite the altered membrane PC composition in the OS (Fig. 5*C*). PDE6β is a rod photoreceptor–specific subunit of PDE that controls cyclic nucleotide-gated channel–mediated cation influx into photoreceptor cells (22). Mice harboring the loss-of-function mutant of PDE6β (termed *rd*1 mouse) showed marked cGMP accumulation and subsequent severe retinal degeneration (23, 24). As *rd*1 mice show similar phenotypes to those of *Lpcat1* KO, early-onset, and light-independent retinal degeneration (21), we assessed whether the altered membrane lipid composition in *Lpcat1* KO photoreceptor cells leads to PDE6β dysfunction. However, cGMP levels in the retina were slightly decreased rather than increased in *Lpcat1* KO mice compared to control mice (Fig. 5*D*). Since the accumulated cGMP-dependent increase in calcium ion influx triggers retinal degeneration in *rd*1 mice, the underlying mechanisms of retinal degeneration in *Lpcat1* KO mice differ from those in PDE6β mutated mice (*rd*1).

### Increased mitochondrial oxidative stress in *Lpcat1* KO mice

**Figure 6:**
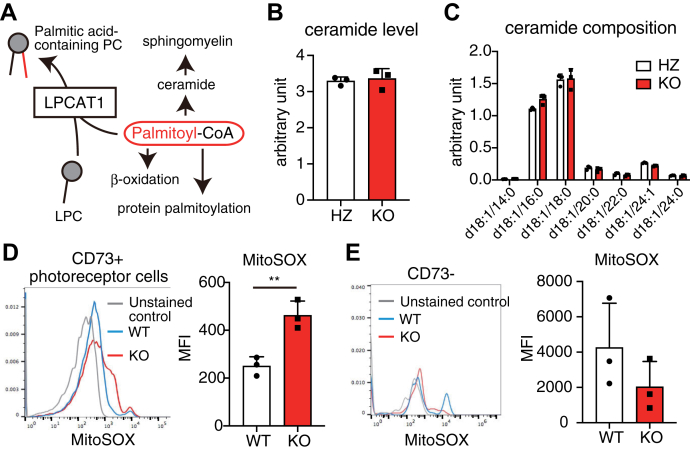
**Increased mitochondrial superoxide production in *Lpcat1* knockout (KO) photoreceptor cells.***A*, intracellular metabolic pathways of palmitoyl-CoA. *B* and *C*, total area values of ceramides (*B*) and ceramide composition (*C*) in P11 *Lpcat1* heterozygous (HZ) and littermate KO retina (n = 3 for each group). *B*, the sum of area values of ceramides were normalized by protein amount and the area values of the internal standard (d18:1/12:0-ceramide) (represented as units/pmol internal standard/μg protein). Data are shown as mean + SD of independent experiments. *D* and *E*, analysis of mitochondrial superoxide production by 3-week-old *Lpcat1* KO and littermate wildtype (WT) photoreceptor cells (*D*) or retinal cells other than photoreceptor cells (*E*) (n = 3 for each group). Retinal cells were stained with CD73 (photoreceptor cells) and MitoSOX Red (mitochondrial superoxide). Data are shown as mean + SD of independent experiments. Significance is based on the unpaired *t* test (∗∗*p* < 0.01). MFI, mean fluorescence intensity.

Excess SFA induces apoptosis (25). Similarly, stearoyl-CoA desaturase (SCD), which converts saturated fatty acyl-CoA to monounsaturated fatty acyl-CoA, protects cells from SFA-induced cell death (26). Together with the significant decrease in the PC 32:1/PC 32:0 ratio (Fig. S4*A*) and SCD mRNA (Fig. S4*B*) during photoreceptor maturation, mature photoreceptor cells are presumably less potent in reducing the intracellular SFA levels by the unsaturation. We hypothesized that SFA stress is involved in photoreceptor-specific apoptosis in *Lpcat1* KO mice based on these results. Several factors, including ceramide accumulation, mitochondrial ROS production, and ER stress, trigger SFA-induced apoptosis (27, 28, 29, 30). Since our transcriptomic analyses showed no sign of increased ER stress in *Lpcat1* photoreceptor cells (Fig. S3*E*), we postulated that the altered palmitoyl-CoA flux (Fig. 6*A*) by *Lpcat1* deletion might be related to retinal degeneration.

To this end, we determined whether the *Lpcat1* deletion leads to ceramide accumulation in the retina. A slight increase in sphingomyelin (SM) suggested that loss of *Lpcat1* increased palmitoyl-CoA availability for sphingolipid synthesis. However, we could not find any clear differences in ceramide levels and compositions between genotypes (Figs. 4*C* and 6, *B* and *C*).

Finally, we explored the possibility that the *Lpcat1* deletion leads to increased mitochondrial FA β-oxidation and consequent ROS accumulation in mature photoreceptor cells. We analyzed the mitochondrial ROS accumulation in retinal cells of P21 control and *Lpcat1* KO mice. Flow cytometric analyses of retinal CD73-positive (photoreceptor) cells showed that the ROS accumulated photoreceptor cells in *Lpcat1* KO mice were increased compared to the control mice (Figs. 6*D* and S5, *A*–*C*). Consistent with TUNEL staining, we observed no differences in ROS accumulation between *Lpcat1* KO and control CD73-negative cells (Fig. 6*E*). Additionally, intracellular ROS levels were also increased in *Lpcat1* KO photoreceptor cells (Fig. S6, *A*–*E*). These results suggest that mitochondrial ROS accumulation triggered by *Lpcat1* deletion is a photoreceptor-specific phenomenon contributing to apoptosis induction.

## Discussion

Here, we demonstrated that the loss of *Lpcat1* leads to an early onset of severe retinal degeneration, triggered by light stimulus–independent photoreceptor cell apoptosis. Although photoreceptor cell damage induces various types of retinal degeneration, the molecular basis of photoreceptor cell death is not fully understood. Our present study suggests that *Lpcat1* deletion–triggered disruption of palmitoyl-CoA flux leads to mitochondrial ROS accumulation and denaturation in photoreceptor cells.

FA saturation of membrane phospholipids affects membrane fluidity and flexibility through their biophysical properties (31, 32). A recent study showed that PUFA-containing phospholipids, especially in DHA-containing phospholipids, are enriched in the center of photoreceptor discs. In contrast, SFA-containing species are enriched in the rim region (33). Therefore, significant levels of saturated PCs in the OS membrane suggest that the photoreceptor OS membrane and/or OS-localized protein require the saturated PC–enriched membrane for their normal structure and functions. In the case of DHA-containing phospholipids, it has been reported that the dramatic decrease in these phospholipids in 1-acylglycerol-3-phosphate *O*-acyltransferase 3, also known as LPLAT3 (4), lysophosphatidic acid acyltransferase 3, KO retina cause the collapse of photoreceptor discs, suggesting the importance of the FA composition of photoreceptor discs in maintaining their structures (13). However, in contrast, the structure of *Lpcat1* KO photoreceptor discs appeared to be normal, regardless of a significant decline in DPPC and PC 30:0. Thus, an increased proportion of PC 34:0, another type of SFA-containing PC, in *Lpcat1* KO photoreceptor OS, may play redundant roles, at least in the formation and/or maintenance of OS disc structures. Conversely, cGMP levels in *Lpcat1* KO retinas were lower than those in WT mice (Fig. 5*D*). Although the decreased cGMP levels in the *Lpcat1* KO retina may reflect the lower number of photoreceptor cells in these mice, it is also possible that the PDE6β activity was affected by altered PC composition in the photoreceptor OS membrane. The detailed mechanisms of this observation should be clarified in future studies.

We demonstrated that mitochondrial ROS accumulation was observed in *Lpcat1* KO photoreceptor cells. This suggests that they are highly susceptible to mitochondrial ROS; therefore, mitochondrial FA oxidation should be strictly regulated. A recent study revealed that photoreceptor cells depend largely on FA oxidation in the mitochondria for ATP production (16, 34). Together with the fact that mature photoreceptor cells require high levels of ATP to maintain the depolarized state in darkness using ATP-dependent channels (35), LPCAT1-mediated incorporation of saturated FAs into PC may be required not only for the production of desaturated PCs but also for the proper control of mitochondrial FA oxidation. Since knockdown of *Lpcat1* expression leads to tumor cell death (9), *Lpcat1*-dependent cell survival of mature photoreceptor cells and cancer cells may show some metabolic similarities.

SCDs are also related to controlling cellular SFA levels by changing saturated acyl-CoA to monounsaturated fatty acyl-CoA (36). It is reported to protect cells from SFA-induced mitochondrial ROS accumulation and apoptosis by changing the fate of SFA from mitochondria to lipid droplets (37, 38). Therefore, a dramatic decrease in SCD expression during photoreceptor maturation, which is reflected in the decreased PC 32:1/PC 32:0 (DPPC) ratio, may also be associated with the sensitivity of *Lpcat1* deletion–triggered increase in palmitoyl-CoA availability. It is also possible that altered mitochondrial membrane lipid composition directly affects mitochondrial functions, such as electron transfer, permeability, and fusion/fission. Additionally, we could not exclude the possibility that mitochondrial denaturation and ROS accumulation result from photoreceptor apoptosis. Therefore, further studies are required to decipher the molecular mechanisms of mitochondrial ROS accumulation in *Lpcat1* KO photoreceptor cells.

From the transcriptomic analyses, we identified the apparent induction of FosB mRNA in the *Lpcat1* KO retina during the progression of retinal degeneration. Although the exact roles of FosB in the retina are unclear, it has been reported that *Fosb* is induced in the retina during retinal degeneration (39). As the onset of photoreceptor cell death was followed by the upregulation of *Fosb* in *Lpcat1* KO mice, this might not be the cause of retinal degeneration. However, considering the role of FosB as a component of activator protein-1, a dimeric transcription factor involved in inflammation, angiogenesis, and apoptosis (40, 41), our results suggest that *Fosb* inhibition may be involved in retinal degeneration in *Lpcat1* KO mice. Importantly, the upregulation of *Fosb* is also found in the monocytes of patients with age-related macular degeneration (42). Therefore, cell types that upregulate *Fosb* during retinal degeneration in *Lpcat1* KO retinas will be clarified by single-cell RNA sequencing in a future study.

In summary, we presented a detailed analysis of *Lpcat1* deletion–induced retinal degeneration. Our data suggest that LPCAT1 is required not only for SFA-containing PC production but also for regulating intracellular ROS levels in photoreceptor cells. Since increasing evidence indicates that the dysregulation of FA metabolism is demonstrated to involve in the various retinal diseases (35, 43), LPCAT1-dependent saturated FA metabolism may also be implicated in human retinal disorders. Therefore, our data provide further insights into the mechanisms underlying photoreceptor cell death–triggered retinal degeneration.

## Experimental procedures

### Animals

All animal experiments were approved and performed following the guidelines of the Animal Research Committee of the National Center for Global Health and Medicine (12053, 13009, 14045, 15037, 16062, 17054, 18046, 19039, 20037, and 21083).

### Mice

*Lpcat1* KO mice (C57BL6/N background) generated in our previous study were used in this study (7). C57BL6/N mice were reported to show retinal degeneration because they harbor a mutation in *the Crumbs homolog 1* (*Crb1*) gene, termed *rd*8 (24, 44). Thus, we crossed *Lpcat1* KO mice with C57BL6/J mice to remove the *rd*8 mutation. Mice without the *rd*8 homozygous mutation were used in this study. Mice were reared in a normal 12-h light–dark cycle or complete darkness from P2 to P9.

### Histological analysis of mouse retina

For frozen sections, enucleated eyes were fixed in 4% paraformaldehyde for 2 h and washed three times with phosphate-buffered saline (PBS) for 5 min, followed by incubation in 10% sucrose/PBS for 3 h, and then in 25% sucrose/PBS overnight at 4 °C. Afterward, the eyes were flash-frozen in a frozen section compound (Leica). Frozen tissue samples were sectioned at 12 μm using a cryostat. For paraffin sections, enucleated eyes, lungs, and livers were fixed in 4% paraformaldehyde at 4 °C for <24 h and then embedded in paraffin. Paraffin-embedded tissue samples were sectioned at 3 μm using a microtome. Tissue sections were stained with hematoxylin-eosin and analyzed using ImageJ software to measure ONL thickness. TUNEL staining was performed (TaKaRa Biochemicals) to detect apoptotic cells in the retina. For immunohistochemical analyses of PDE6β and rhodopsin, rabbit polyclonal PDE6β (ab5663; Abcam) and 1D4 (ab3424; Abcam) antibodies were used, respectively. Immunohistochemical analyses were performed using the VECTASTAIN Elite ABC kit (Vector Laboratories).

### Quantitative reverse transcriptase PCR

Total RNA was extracted from the retina or isolated photoreceptor cells using an RNeasy Mini Kit (Qiagen). Single-strand cDNA was synthesized using SuperScript III reverse transcriptase (Thermo Fisher Scientific). Using random primers (Thermo Fisher Scientific), qPCR was performed using the Fast SYBR Green Master Mix (Applied Biosystems) with the StepOnePlus real-time PCR system (Applied Biosystems). Relative mRNA expression levels were calculated using the comparative cycle threshold method and normalized to GAPDH mRNA levels. Primers for qPCR were as follows: gene name: forward primer (5′-3′), reverse primer (5′-3′), and amplicon size (bp)—*Gapdh*, TGACAATGAATACGGCTACAGCA, CTCCTGTTATTATGGGGGTCTGG (188) *Lpcat1*; TTCCCTGGGACCTCCTGATAAGG, GAGGAATGTGGAGCCAAGGTGAG, (186). *Rhodopsin*; CTCTTCATGGTCTTCGGAGGATT, GTGAAGACCACACCCATGATAGC, (230). *Fosb*; AGGCAGAGCTGGAGTCGGAGAT, GCCGAGGACTTGAACTTCACTTCG, (151). *Pkc*α: GTGTCCTACCCCAAATCCTTGTC, GTTGGATCTCCCTGTTCTCCAGT (172) *Pax6*; GGCGACTCCAGAAGTTGTAAGCA, GTTGCTTTTCGCTAGCCAGGTTG, (173). *Cd86*; TGTTTCCGTGGAGACGCAAG, TTGAGCCTTTGTAAATGGGCA, (70). *Egr1*: CAACCCTATGAGCACCTGACCAC, GTCGTTTGGCTGGGATAACTCGT (95) *Egr2*; GCCAAGGCCGTAGACAAAATC, CCACTCCGTTCATCTGGTCA, (154). *Atf3*; TTTGCTAACCTGACACCCTTTG, AGAGGACATCCGATGGCAGA, (81). *Jun*; CCTTCTACGACGATGCCCTC, GGTTCAAGGTCATGCTCTGTTT, (102). *Junb*; TCACGACGACTCTTACGCAG, CCTTGAGACCCCGATAGGGA, (125). *Rpe65*; AGTTCCCCTGCAGTGATCGTTTC, ACCCCCATGCTTTCATTGGACTC, (178). *Ddit3*; CTGAGTCCCTGCCTTTCACCT, GCAGGGTCAAGAGTAGTGAAGGTTT, (166). *Scd*; ACTGGTTCCCTCCTGCAAG, GTGATCTCGGGCCCATTC, (199).

### Microsomal fraction preparation

The retina was homogenized in lysis buffer (100 mM Tris-HCl [pH 7.4], 300 mM sucrose) containing a 1× cOmplete protease inhibitor cocktail (Roche, Switzerland). After removing the tissue debris (800*g*, 10 min, 4 °C), microsomal fractions were collected by ultracentrifugation (100,000*g*, 60 min, 4 °C). The pellets (microsomal fractions) were resuspended in ice-cold buffer containing 20 mM Tris-HCl (pH 7.4), 300 mM sucrose, and 1 mM EDTA. Protein concentrations were measured by the Bradford method using a protein assay (Bio-Rad).

### LC-MS/MS

For PC analyses, lipids were extracted from microsomal fractions or retinal OS using the Bligh & Dyer method (45). Subsequently, the extracted lipids were dried using a centrifugal evaporator and reconstituted in methanol. Lipid samples were then analyzed by LC-electrospray ionization-MS/MS with multiple reaction monitoring (MRM). LC-MS/MS analyses were performed using a Nexera UHPLC system and triple quadrupole mass spectrometer LCMS-8050 (Shimadzu Corp). The lipid samples (5 μl/injection) were separated on an Acquity UPLC BEH C8 column (1.7 μm, 2.1 × 100 mm, Waters) at a flow rate of 0.35 ml/min with a gradient of mobile phase A (5 mM NH_4_HCO_3_/water), B (acetonitrile), and C (isopropyl alcohol). The column oven temperature was set to 47 °C. The gradient setting was as follows—time (%A/%B/%C): 0 min (50/45/5), 10 min (20/75/5), 20 min (20/50/30), 27.5 min (5/5/90), 28.5 min (5/5/90), 28.6 min (50/45/5) for retina, and 0 min (75/20/5), 20 min (20/75/5), 40 min (20/5/75), 45 min (5/5/90), 50 min (5/5/90), 55 min (75/20/5) for the retinal OS. PC species were detected in positive-ion mode following MRM transitions: (Q1, Q3), ([M + H]^+^, 184.1). The FA composition of PC is denoted as Z XX:YY (where Z indicates the lipid class). XX and YY indicate the sum of the carbon numbers and double bonds, respectively.

For the analysis of ceramides, lipids were extracted from the mouse retina as previously described, with slight modifications (46). Briefly, retinas were homogenized in lysis buffer (100 mM Tris-HCl [pH 7.4], 300 mM sucrose) containing a 1× cOmplete protease inhibitor cocktail (Roche). After removing the tissue debris (800*g*, 10 min, 4 °C), 20 μl of lysate was mixed with 30 μl of PBS, 50 μl of d18:1/12:0-ceramide (50 μM in methanol), 750 μl of *n*-butanol, 30 μl of phosphate buffer (500 mM, pH 5.8), and 170 μl of water. The samples were then vortexed for 5 min, sonicated for 3 min in a bath sonicator, and centrifuged at 1000*g* for 5 min at 4 °C. An upper phase (700 μl) of the first butanol extract was transferred to a new tube. Three hundred fifty microliters of ethyl acetate and 350 μl of hexane were added to the remaining lower phase for the second extraction. After vortexing for 5 min at 4 °C, 700 μl of the upper phase (second extract) was combined with the first butanol extract and added to 700 μl of methanol. The combined lipid extract (210 μl,10%) was dried using a centrifugal evaporator, reconstituted in 60 μl of solvent B (isopropyl alcohol with 0.2% formic acid and 0.028% ammonia), and then mixed with 90 μl of solvent A (isopropyl alcohol/methanol/water [5:1:4] with 0.2% formic acid, 0.028% ammonia, and 5 μM phosphoric acid). The samples were analyzed by LC-MS/MS with selected reaction monitoring. LC-MS/MS analysis was performed using an AQUITY UPLC system and a TSQ Vantage triple-stage quadrupole mass spectrometer. The extracted lipids (5 μl/injection) were separated on an Acclaim PepMap 100 C18 column (3 μm, 1.0 × 150 mm, Thermo Fisher Scientific) at a flow rate of 0.045 ml/min with a linear gradient of solvent A and solvent B. The column temperature was 45 °C. The gradient transition was as follows: time (%A/%B): 0 min (70/30), 2 min (50/50), 12 min (20/80), 12.5 min (5/95), 22.5 min (5/95), 23 min (95/5), 24 min (95/5), 26 min (70/30), 28 min (70/30). Ceramide species were detected in positive-ion mode following MRM transitions (Q1, Q3), ([M − H_2_O + H]^+^, 264.3 or 266.3).

### Microarray

Total RNA from the whole retina or isolated photoreceptor cells was extracted using the RNeasy Mini Kit (Qiagen). Total RNA was examined using the SurePrint G3 Mouse GE 8 × 60K Microarray (Agilent Technologies). Data were quantified using the Agilent Feature Extraction software (Agilent Technologies) and normalized by 75 percentile shift normalization using GeneSpring software (Agilent Technologies). The list of highly expressed genes in *Lpcat1* KO than in control with fold change >1.2 or 1.5 and q-value <0.05 or 0.5 was analyzed for common functions of altered genes using gene ontology (GO) terms using the Database for Annotation, Visualization, and Integrated Discovery.

### Isolation of photoreceptor cells

Photoreceptor cells were isolated from the mouse retina as previously described, with slight modifications (18, 47). Retinas were isolated from *Lpcat1* HZ and KO mice and dissociated in 500 μl of 0.25% trypsin (Nacalai Tesque) in PBS for 15 min at 37 °C. Then, 500 μl of 20% fetal bovine serum (Thermo Fisher Scientific) and 1 μl DNase I (Invitrogen) were added on ice. Mechanical dissociation was performed by pipetting 20 times using a 1-ml tip. The cells were collected by centrifugation at 300*g* for 5 min. The retinal cell pellet was washed in 700 μl of 2% bovine serum albumin (BSA)/PBS and centrifuged at 300*g* for 5 min. The cell pellet was resuspended in 50 μl of 2% BSA/PBS, and photoreceptor cells were labeled with PE rat anti-mouse CD73 antibody (TY/23, BD Pharmingen) for 30 min at 4 °C. Cells were washed with 700 μl of 2% BSA/PBS and centrifuged at 300*g* for 5 min. The cell pellet was resuspended in 80 μl of 2% BSA/PBS and incubated with 20 μl of anti-PE MicroBeads UltraPure (Miltenyi Biotec) for 15 min at 4 °C. After washing with 700 μl of 2% BSA/PBS and centrifugation at 300*g* for 5 min, the cell pellet was resuspended in 500 μl of 2% BSA/PBS and filtered through a 35-μm preseparation filter (Corning). The CD73-positive cells were collected as photoreceptor cells using an autoMACS Pro Separator (Miltenyi Biotec).

### Retinal OS isolation

Mouse retinal OS was purified as described previously (48). Twelve mouse retinas were suspended in 120 μl of 8% OptiPrep (Abbott Diagnostics Technologies AS) in Ringer’s buffer (130 mM NaCl, 3.6 mM KCl, 2.4 mM MgCl_2_, 1.2 mM CaCl_2_, 0.02 mM EDTA, 10 mM Hepes-NaOH, pH7.4). Retinal OS fractions were separated from the retina by vortexing for 1 min. The samples were then centrifuged at 200*g* for 1 min. The vortexing and sedimentation sequence was repeated six times to collect the remaining retinal OS fraction in the retinal pellets. The collected supernatants (retinal OS fractions) were combined (600 μl) and overlaid onto a discontinuous gradient of OptiPrep (Veritas) (10%, 18%, 24%, and 30%) in Ringer’s buffer (1.8 ml each) and centrifuged at 25,900*g* for 30 min. After centrifugation, the retinal OS fraction (approximately two-thirds of the way from the top of the gradient) was harvested and diluted three times with Ringer’s buffer. The contaminated retinal fractions were removed by centrifugation at 500*g* for 3 min. The supernatant (pure retinal OS fraction) was precipitated by centrifugation at 26,500*g* for 30 min. The resultant retinal OS pellets (microsomal fractions) were resuspended in ice-cold buffer containing 20 mM Tris-HCl (pH 7.4), 300 mM sucrose, and 1 mM EDTA.

### Determination of cGMP levels

P10 mice were dark-adapted for 16 h, and retinas were dissected under dim red light and frozen in liquid nitrogen. Two frozen retinas were homogenized in 200 μl of 5% trichloroacetic acid/water under dim red light and centrifuged at 1500*g* for 10 min. The resultant supernatant was used for cGMP measurements. cGMP levels were determined using a cyclic GMP EIA kit (Cayman) according to the manufacturer’s instructions.

### Transmitted electron microscopy

Enucleated eyes were immediately cut into hemispheres in a fixative containing 2% paraformaldehyde and 2.5% glutaraldehyde. The samples were then fixed in the same fixative for 2 h, followed by an additional fixation in the buffer containing 2% OsO4. The samples were dehydrated in a graded series of ethanol (70%-90%-100%) and embedded in an Epon 812 resin mixture (TAAB Laboratories Equipment Ltd). Ultrathin sections were created using an ultramicrotome, stained with uranyl acetate and lead citrate, and observed under an electron microscope (Hitachi H-7500).

### Detection of mitochondrial ROS

ROS production was measured by flow cytometry using MitoSOX Red (Invitrogen). Retina was isolated from 3-week-old *Lpcat1* WT and KO mice and dissociated in 500 μl of 0.25% trypsin (Nacalai Tesque) in PBS for 15 min at 37 °C. Then, 500 μl of 20% fetal bovine serum (Thermo Fisher Scientific) and 1 μl DNase I (Invitrogen) were added on ice. Mechanical dissociation was performed by pipetting 20 times using a 1-ml tip. The cells were collected by centrifugation at 300*g* for 5 min. The retinal cell pellet was washed in 700 μl of 2% BSA/PBS and centrifuged at 300*g* for 5 min. The cell pellets were resuspended in 2% BSA/PBS. Photoreceptor cells were labeled with FITC anti-mouse CD73 antibody (TY/11.8, BioLegend). Dead cells were stained with LIVE/DEAD Fixable Far Red Dead Cell Stain kit (Invitrogen), and retinal cells were stained with 5 μM MitoSOX Red for 15 min at 37 °C. The cells were centrifuged at 300*g* for 5 min. The cell pellet was resuspended in 500 μl of 2% BSA/PBS and filtered through a 35-μm preseparation filter (Corning). The fluorescence intensity was measured using a BD Accuri flow cytometer (BD Biosciences).

### Statistical analysis

Unpaired t-tests were used when the two groups were compared. When two factors were present, a two-way ANOVA test was performed. Bonferroni’s post hoc test was used when the ANOVA showed significance. All statistical analyses were performed using GraphPad Prism9 (version 9.2.0).

## Data availability

The microarray data are deposited at GEO: GSE184815 (2-week-old retina). The microarray data are deposited at GEO: GSE184817 (P8 photoreceptor cell).

## Supporting information

This article contains supporting information (45, 49).

## Conflict of interest

The Department of Lipid Signaling, National Center for Global Health and Medicine, is financially supported by 10.13039/501100013170ONO Pharmaceutical Co, Ltd, Japan.
