# Supplementary material for: Lysophosphatidylcholine acyltransferase 1 controls mitochondrial reactive oxygen species generation and survival of retinal photoreceptor cells
Source: J Biol Chem. 2022 Apr 20;298(6):101958. doi: 10.1016/j.jbc.2022.101958 (PMC9136105; doi:10.1016/j.jbc.2022.101958)
Supplement: Supplemental Figures S1–S6 and Table S1 [file mmc1.pdf]

Supporting information

Lysophosphatidylcholine acyltransferase 1 controls mitochondrial reactive oxygen species generation and survival of the retinal photoreceptor cells

Katsuyuki Nagata, Daisuke Hishikawa, Hiroshi Sagara, Masamichi Saito, Sumiko Watanabe, Takao Shimizu, and Hideo Shindou

The supporting information includes supporting experimental procedures, six supplementary figures and one supplementary table.

Supporting Experimental Procedures:

Immunoblot:

Protein samples were resolved on 4% / 10% SDS-polyacrylamide gels and transferred to nitrocellulose membranes (GE Healthcare, USA.) using a Trans-Blot Turbo (Bio-Rad, USA). Membranes were blocked with blocking buffer (5% skim milk in Tris-based buffer with 0.1% polyoxyethylene(20) sorbitan monolaurate (Wako, Japan)) at room temperature for 1 h. The membrane was incubated with rabbit-anti-LPCAT1 antibody (1) or mouse-anti-β-actin (GE Healthcare) at 4°C for 16 hours. The membrane was washed three times with wash buffer (Tris-based buffer with 0.1% polyoxyethylene(20) sorbitan monolaurate) for 5 min, and incubated with anti-rabbit IgG antibody conjugated to horseradish (GE Healthcare, USA.) or anti-mouse IgG antibody conjugated to horseradish (GE Healthcare) at room temperature for 1 h. The membrane was washed three times with wash buffer for 5 min each and developed using ECL reagent (GE Healthcare, USA.). Immunoreactive proteins were visualized using ImageQuant LAS500 (GE Healthcare, USA.).

Measurement of total sphingomyelin (SM) levels:

Retinal lipids were separated using thin layer chromatography (TLC) to determine the total SM levels. Lipids were extracted from the frozen retina as follows: The frozen retinas were sonicated in 200 μL of methanol. Then, the retinal lipids in the methanol suspension were further extracted using the Bligh and Dyer method (2). The extracted lipids were treated with KOH (final 0.125N), followed by 15 min of heating at 60°C. Lipid solutions were spotted (1 μL) onto chromatoplates precoated with silica gel 60 (Merck, Germany). The developing solvent system used in the TLC experiment was chloroform: methanol: acetic acid: water (25:20:4:2, v/v/v/v). Lipid spots were detected with primulin and then visualized using ImageQuant LAS500 (GE Healthcare, USA.). SM was identified by comparison with the spot of authentic SM (860062; Avanti Polar Lipids, Inc., USA.). The signal intensity of SM was quantified using Image J software.

Detection of intracellular reactive oxygen species (ROS) generation:

Intracellular ROS generation was measured by flow cytometry using the Highly Sensitive DCFH-DA ROS Assay Kit (Dojindo, Japan). Retina was isolated from 3-week-old *Lpcat1* WT and KO mice and dissociated in 500 μL of 0.25% trypsin (Nacalai Tesque, Japan) in PBS for 15 min at 37°C. Then, 500 μL of 20% fetal bovine serum (Thermo Fisher Scientific, USA.) and 1 μL DNase I (Invitrogen, Germany) were added on ice. Mechanical dissociation was performed by pipetting 20 times using a 1 mL tip. The cells were collected by centrifugation at 300 × *g* for 5 min. The retinal cell pellet was washed in 700 μL of 2% BSA/PBS and centrifuged at 300 × *g* for 5 min. The cell pellets were resuspended in 2% BSA/PBS. Photoreceptor cells were labeled with PE rat anti-mouse CD73 antibody (TY/23, BD Pharmingen, USA.). Dead cells were stained with LIVE/DEAD Fixable Far Red Dead Cell Stain kit (Invitrogen, Germany), and retinal cells were treated with highly sensitive DCFH-DA dye for 30 min at 37°C. The cells were centrifuged at 300 × *g* for 5 min. The cell pellet was resuspended in 500 μL of 2% BSA/PBS and filtered through a 35-μm pre-separation filter (Corning, USA.). The fluorescence intensity was measured using a BD Accuri flow cytometer (BD Biosciences, USA.).

References:

1. Harayama, T., Shindou, H., Ogasawara, R., Suwabe, A., and Shimizu, T. (2008) Identification of a novel noninflammatory biosynthetic pathway of platelet-activating factor. *J Biol Chem* **283**, 11097-11106
2. Bligh, E. G., and Dyer, W. J. (1959) A rapid method of total lipid extraction and purification. *Can J Biochem Physiol* **37**, 911-917

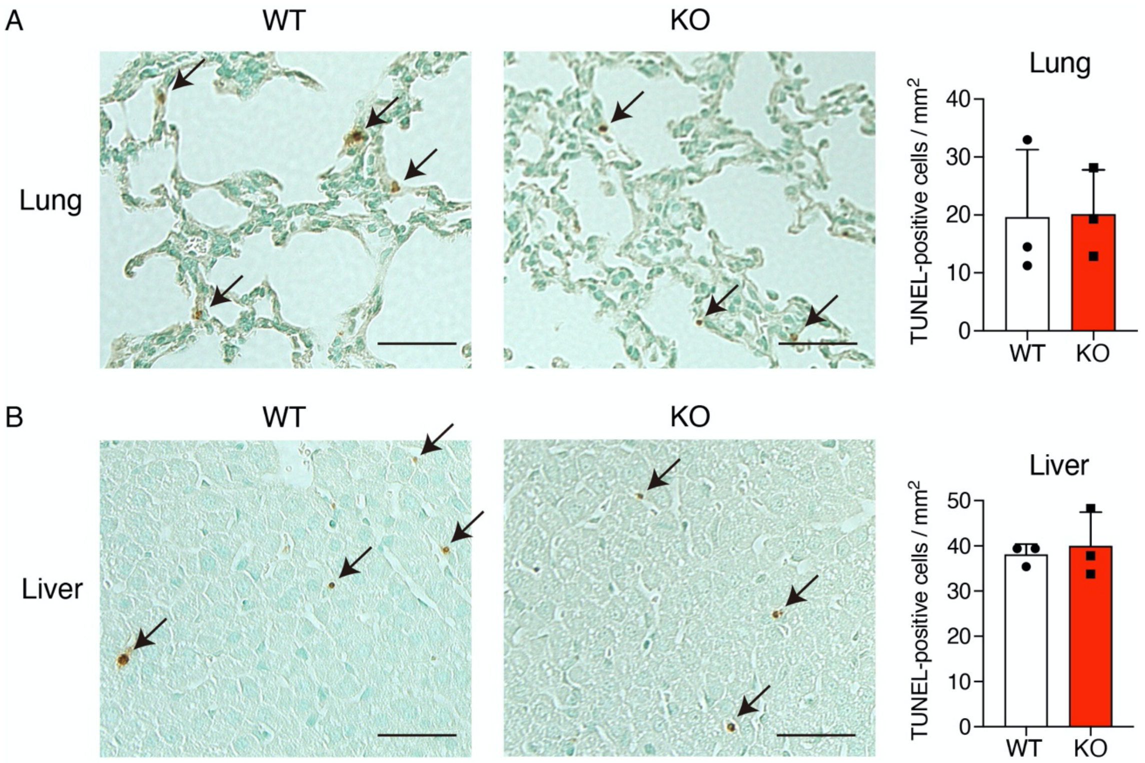

Figure S1. Apoptotic cells of *Lpcat1* wild-type (WT) and knockout (KO) lung and liver. Representative images of terminal deoxynucleotidyl transferase dUTP nick-end labeling (TUNEL) staining of 2-week-old *Lpcat1* KO and littermate WT lung (A) and liver (B). Nuclei were stained with Methyl Green. Arrows indicated TUNEL-positive nuclei, which is detected by 3,3'-diaminobenzidine (DAB, shown in brown). Representative images from three independent experiments were shown. Right bar graphs showed the average number of TUNEL-positive cells per 1 mm^2 in randomly selected areas. Data are shown as mean + SD of independent experiments (n=3 for each group). Scale bars are 50 μm.

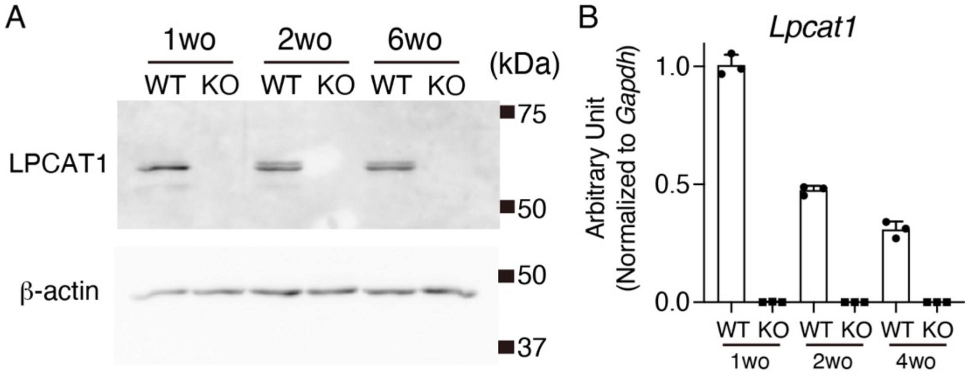

Figure S2. The expression of *Lpcat1* mRNA and protein during retinal maturation in *Lpcat1* wild-type (WT) and knockout (KO) retina. A, Representative image of immunoblot analysis of the amount of LPCAT1 in 1-, 2- and 6-week-old (wo) *Lpcat1* wild-type (WT) and knockout (KO) retina. Similar results were obtained in three independent experiments. β-actin was used as a loading control. B, *Lpcat1* mRNA expression in 1-, 2- and 4-week-old *Lpcat1* WT and KO retina. The expression level was normalized by the glyceraldehyde 3-phosphate dehydrogenase (*Gapdh*) gene. Data are shown as mean + SD of independent experiments (n=3 for each group).

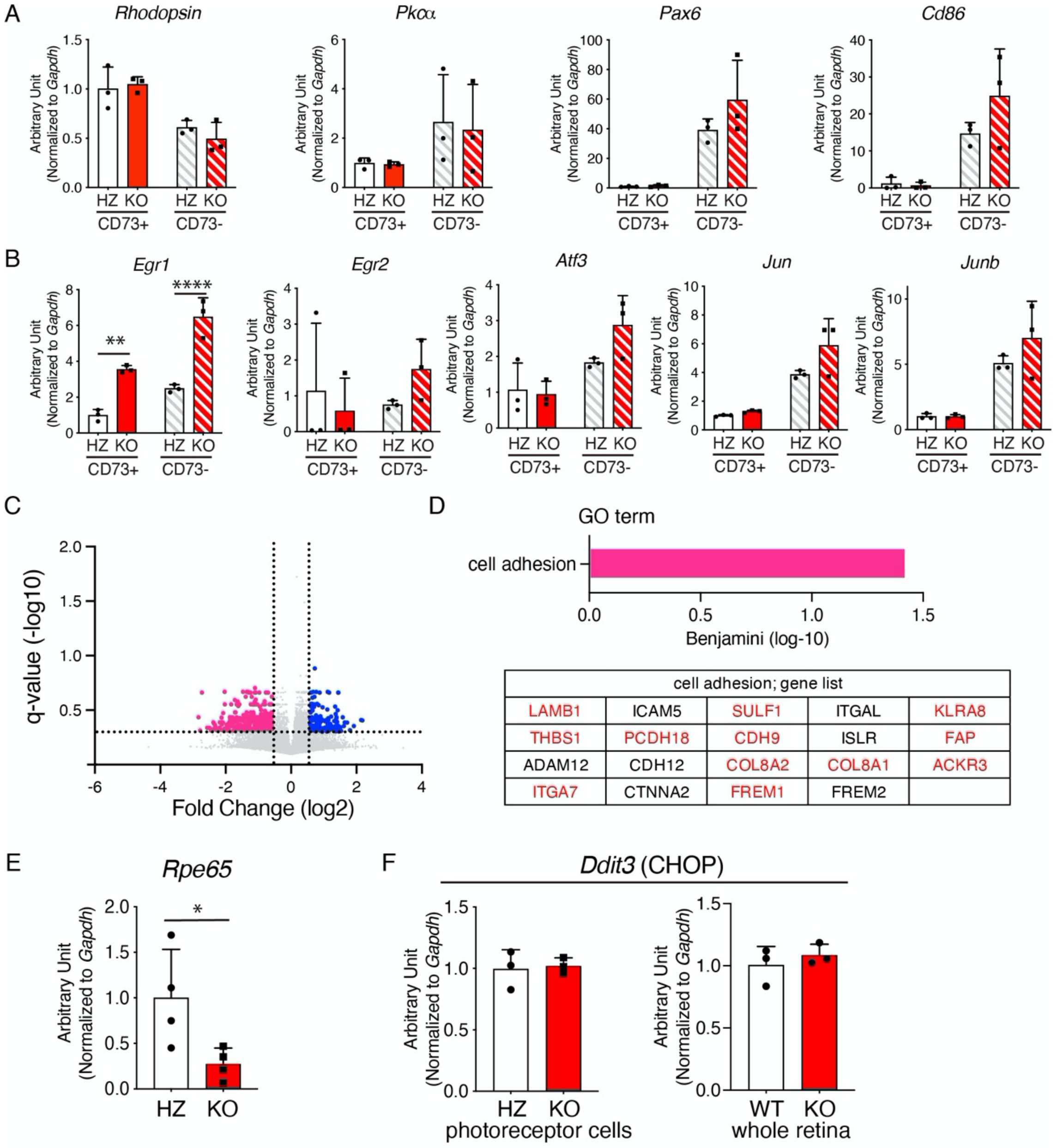

Figure S3. Gene expression analyses of *Lpcat1* knockout (KO) retinal cells. A, Cell-type marker gene mRNA expression in isolated CD73-positive (photoreceptor) and -negative cells from postnatal day 14 (P14) *Lpcat1* heterozygote (HZ) and KO retina by qPCR analysis. *Rhodopsin*, *Pkcα*, *Pax6*, and *Cd86* were used as rod photoreceptor cells, bipolar cells, retinal progenitor cells, and glial cells, respectively. Data are shown as mean + SD of independent experiments (n=3 for each group). B, mRNA expression of immediate early genes in P14 *Lpcat1* HZ and KO of CD73-positive (photoreceptor cells) and -negative retinal cells by qPCR analysis. Data are shown as mean + SD of

independent experiments (n=3 for each group). Significance is based on two-way ANOVA followed by Bonferroni's multiple comparisons test (**$P < 0.01$, ****$P < 0.0001$). C and D, Transcriptomic analysis of isolated photoreceptor cells of P8 *Lpcat1* KO and littermate WT mice. C, Volcano plot of differentially expressed genes (DEGs) between P8 *Lpcat1* HZ and KO photoreceptor cells. DEGs (Fold change > 1.5 or < -1.5, q-value < 0.5) are highlighted in blue; increased in *Lpcat1* KO, and magenta; decreased in *Lpcat1* KO (n=4 for each group). D, Functional annotation of downregulated genes in *Lpcat1* KO photoreceptor cells. The gene ontology (GO) term from "biological processes" is shown (Benjamini-corrected $P < 0.05$, upper panel). Genes related to cell adhesion are the only category that is significantly altered. The lower panel shows the list of downregulated genes in *Lpcat1* KO photoreceptor cells, which belongs to GO term of cell adhesion. Genes in red indicate highly expressed genes in RPE, which confirmed by bioGPS, an open-source gene expression database (http://biogps.org/#goto=welcome). E, *Rpe65* mRNA expression in photoreceptor cells of P8 *Lpcat1* KO and littermate HZ mice by qPCR analysis. Data are shown as mean + SD of independent experiments (n=4 for each group). Significance is based on unpaired *t*-test (*$P < 0.05$). F, *Ddit3* (CHOP) mRNA expression in P14 *Lpcat1* HZ and KO photoreceptor cells and P14 *Lpcat1* wild-type (WT) and KO whole retina. Data are shown as mean + SD of independent experiments (n=3 for each group). A, B, E, and F, The expression level was normalized by glyceraldehyde 3-phosphate dehydrogenase (*Gapdh*).

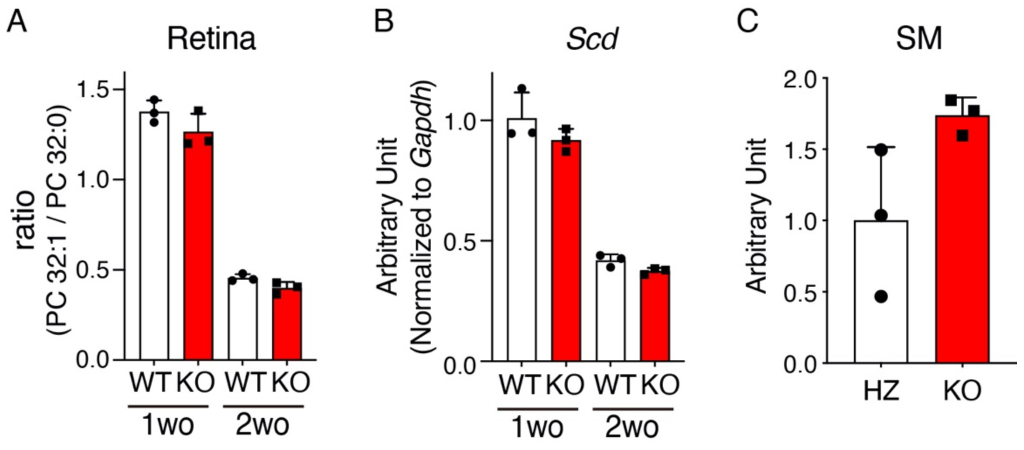

Figure S4. Fatty acid-related factors during retinal maturation. A, Relative ratio of area values of PC 32:1 to PC 32:0 in 1- and 2-week-old (wo) *Lpcat1* wild-type (WT) and knockout (KO) retina. Data are shown as mean + SD of independent experiments (n=3 for each group). B, Gene expression of *Scd* at mRNA level in 1- and 2-week-old *Lpcat1* WT and KO retina. The expression level was normalized by glyceraldehyde 3-phosphate dehydrogenase (*Gapdh*). Data are shown as mean + SD of independent experiments (n=3 for each group). C, Total sphingomyelin (SM) levels in 2-week-old *Lpcat1* heterozygote (HZ) and littermate KO retina. Lipids extracted retinal tissues were separated with thin layer chromatography (TLC) and stained with primulin. The spot of SM was identified using authentic SM. The signal intensity of SM was quantified by Image J software. Data are shown as mean + SD of independent experiments (n=3 for each group).

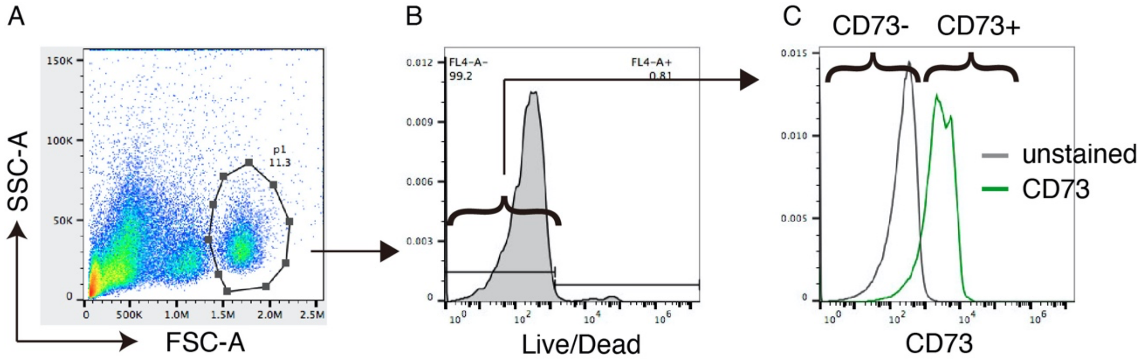

Figure S5. Gating strategy of retinal cells. A-C, Retinal cells were labeled with Live/Dead fluorescent dye and anti-CD73 antibody. A, Retinal cells (inside the indicated gate) were separated from cellular debris by forward versus side scatter (FSC vs SSC) gating. B, Living retinal cells were selected as Live/Dead staining-negative cells. C, Retinal cells were further separated into CD73-positive (photoreceptor) and -negative cells.

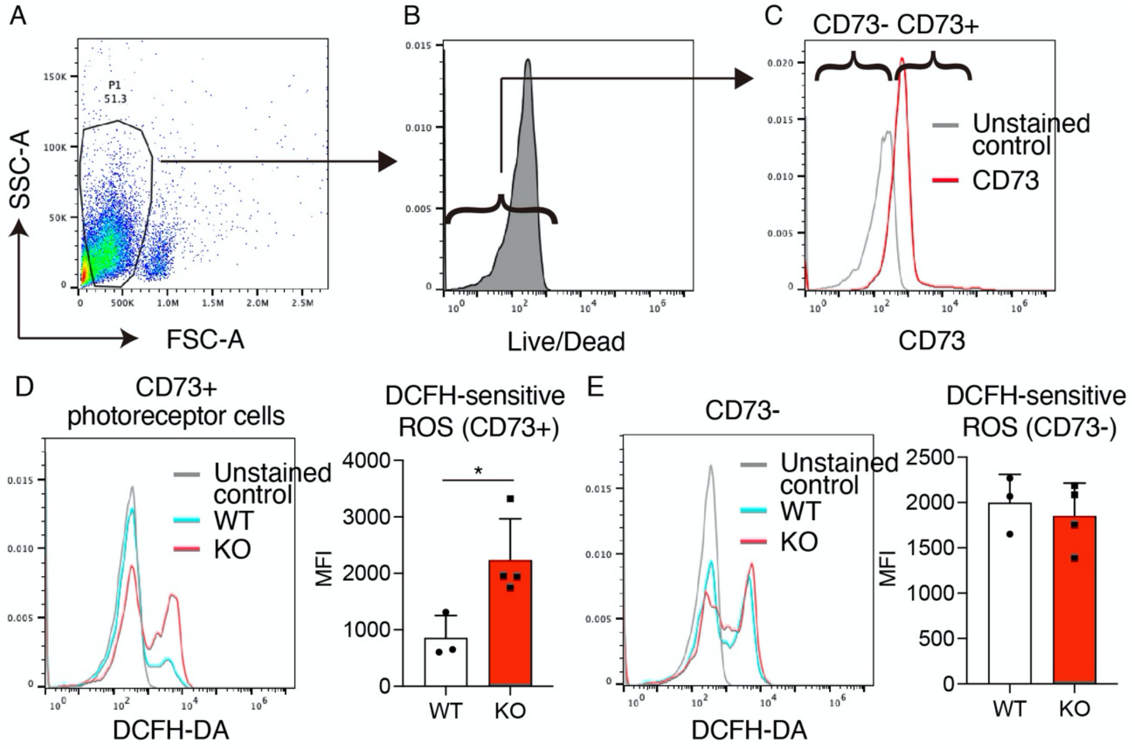

Figure S6. Intracellular ROS levels in isolated photoreceptor cells. A-C, Gating strategy of living retinal CD73-positive and -negative cells of 3-week-old mice. A, Representative forward versus side scatter (FSC vs SSC) plot of retinal cells. Cells within the circle were used as retinal cells. B, Living retinal cells were selected as Live/Dead staining-negative cells. C, Living retinal cells were further separated into CD73-positive (photoreceptor) and -negative cells. D and E, Analysis of intracellular ROS levels in 3-week-old *Lpcat1* knockout (KO) and littermate wild-type (WT) photoreceptor cells (D) or retinal cells other than photoreceptor cells (E) (n=3 for WT, n=4 for KO). Data are shown as mean + SD of independent experiments. Significance is based on unpaired *t*-test (*$P < 0.05$).

symbol	*gene name*	*fold change*
Ccl4	chemokine (C-C motif) ligand 4	5.6
Egr2	early growth response 2, transcript variant 1	4.2
Egr1	early growth response 1	4.1
Mt1	metallothionein 1	4.0
Cst7	cystatin F (leukocystatin)	3.9
Etnppl	ethanolamine phosphate phospholyase, transcript variant 1	3.8
Cebpe	CCAAT/enhancer binding protein (C/EBP), epsilon	3.8
Edn2	endothelin 2	3.8
Fosb	FBJ osteosarcoma oncogene B, transcript variant 1	2.9
Fos	FBJ osteosarcoma oncogene	2.7
Itgax	integrin alpha X	2.6
Nr4a1	nuclear receptor subfamily 4, group A, member 1	2.6
Fam107a	family with sequence similarity 107, member A	2.5
C4b	complement component 4B (Chido blood group)	2.5

Table S1. List of upregulated genes in 2-week-old *Lpcat1* knockout retina. Immediate early genes (IEGs) are shown in red.
